# Ultrasensitive Optical Detection of Water Pressure in Microfluidics Using Smart Reduced Graphene Oxide Glass

**DOI:** 10.3389/fchem.2019.00395

**Published:** 2019-05-31

**Authors:** Wei Xin, Tiange Wu, Tingting Zou, Ye Wang, Wenshuai Jiang, Fei Xing, JianJun Yang, Chunlei Guo

**Affiliations:** ^1^The Guo China-US Photonics Laboratory, State Key Laboratory of Applied Optics, Changchun Institute of Optics, Fine Mechanics, and Physics, Chinese Academy of Sciences, Changchun, China; ^2^School of Physics and Optoelectronic Engineering, Shandong University of Technology, Zibo, China; ^3^School of Biomedical Engineering, Xinxiang Medical University, Xinxiang, China; ^4^The Institute of Optics, University of Rochester, Rochester, NY, United States

**Keywords:** reduced graphene oxide, microfludics, polarization-dependent total internal reflection, ultrasonic waves, water pressure

## Abstract

Despite recent progresses in the field of microfluidics, the effect of liquid pressure on the detection accuracy has been rarely studied. Here, we perform a quantitative analysis of such effect, by utilizing the sensitive optical responses of graphene to the refractive index (RI) change of its surrounding environment. We utilize a reflection coupling configuration by combining the total internal reflection (TIR) and ultrasonic waves. The high-performance graphene is processed on common glasses by using the solution-processable oxidation-reduction method. We find that the RI change of water caused by a pressure as small as 500 Pa generated by the liquid level change in the microfluidics can be measured directly. The detection accuracy and response time limits are approximately 280 Pa and 100 ns, respectively. The Maxwell's boundary conditions, Fresnel's law, and Pascal's law are used in theoretical analyses. This work highlights the importance of liquid pressure in microfluidics and provides guidance in designing and accurate detection of microfluidic devices.

## Introduction

Microfluidic, also called the “lab-on-a-chip,” is an exciting field that offers manageable and sustainable implementation of chemical and biological processes (Mark et al., 2010). Because of the small diameter and the inevitable enlarged contact area of a microfluidic channel, the liquid flow inside possesses different physical properties compared to that in the fluid systems at macroscopic scales (Anna et al., 2003; Sun et al., 2008). The phenomena such as capillary, laminar, and mixture flows should be taken into consideration, which are all closely related to liquid pressure (Cristini and Tan, 2004; Liu et al., 2006). Therefore, especially for highly sensitive microfluidic chips, an in-depth understanding of the influence of liquid pressure on detection is important for the design and testing accuracy. Although great advances have been made in the field of theoretical modeling of liquid flow processes, such as the establishment of Navier-Stokes and Bernoulli's equations, the practicality in complex microfluidic environment will need to be tested (Gallouët et al., 2010). The existence of only theories without experimental verification may cover up the accurate detection of microfluidic systems and the precise control of micro-reactions.

In this work, we developed an effective method for ultrasensitive optical detection of water pressure in a microfluidic chip with high-performance reduced graphene oxide (rGO) on regular glass (Fowler et al., 2009; Zhou et al., 2009). The device works under a reflection coupling configuration by combining the total internal reflection (TIR) and ultrasonic waves operations. This configuration has been proven very sensitive to the refractive index (RI) change of different contacted materials such as gases, liquids, and biomolecules, and therefore be well-suited for the detection of water pressure in microfluidic channels (Robinson et al., 2008; Shao et al., 2010). It can resolve an ultra-small, fast RI change on the order of magnitude 10^−8^, and detect the water pressure as tiny as 500 Pa tuned by changing the liquid level in the microfluidic. We found a linear dependence of the voltage signal on the liquid level. The detection accuracy and response time limits are about 280 Pa and 100 ns, respectively. Moreover, unlike graphene glass prepared through chemical vapor deposition (CVD) method, the microfluidic chip here is based on the solution-processable rGO, which is more suitable for large-scale commercial production without the consideration of apparatus independent, rigorous conditions, fine operations, and complicated transfer processes (Zhang et al., 2011; Badhulika et al., 2015; Chen et al., 2017; Wang et al., 2017; Han et al., 2019). However, our experiments are not limited to rGO alone. If we find more suitable materials considering the chemical stability and wettability, such as other two-dimensional (2D) materials, heterostructures, or composite materials combined with nanowires, quantum dots, etc., we may get more extensive measurements information. This study highlights the importance of liquid pressure effects in analyzing and optimizing microfluidic devices and opens up potential applications.

## Materials and Methods

### Preparation of Graphene Oxide (GO)

Crystalline flake graphite (99.99% purity, Laixi Baichuan Graphite Co. Ltd.) was used as the raw material for graphene oxide preparation with modified Hummers method (Hummers and Offeman, 1958; Becerril et al., 2008). First, 5 g of graphite and 3.5 g of NaNO_3_ were placed in flask. In the second step, 250 ml concentrated H_2_SO_4_ was added in an ice-water bath with continuous stirring, and 25 g KMnO_4_ was added later within 1 h and stirred continuously at room temperature for 7 days. After that, 250 ml pure water was added and stirred for 1.5 h. The subsequent water-bath heating should be maintained at 80°C for 3 h. 250 ml pure water was then injected in the flask again. The mixture was transferred into a 1,000 ml beaker when the temperature was reduced to room temperature, And then 25 ml of H_2_O_2_ were needed to add and stirred for 1–2 h to remove the generated impurities. The repetition of pickling, centrifugation, and filtration of the supernatant was also needed to make purification of graphene oxide until the centrifugation without stratification. At last, the GO aqueous solution was dried at low temperature of −40°C to obtain the powdered one (Xing et al., 2016).

### Preparation of rGO Glass

At first, the common glasses were polished with a high precision polisher to achieve a smooth optical surface, followed by an ultrasonic cleaning in water, and organic solvents for 15 min. After drying in nitrogen gas, the glasses were exposed to oxygen plasma at a power of 20 W for 2 min. This hydrophilic treatment is necessary for a uniform deposition of GO solution thereon. Then the GO glasses were obtained by dripping the aforementioned GO solution (2 mg/ml) on the glasses and spin-coating at 1,500 r/min for 50 s following by naturally dried at room temperature. The thickness of GO film can be changed by repeating this step. After thermal reduction in CVD vacuum system (HTF55347C-1, Thermo Fisher Scientific Co. Ltd.) in H_2_/Ar (5%/95%) shielding gas at 450°C for 4 h, the rGO glasses were obtained (McAllister et al., 2007; Pei and Cheng, 2012). Finally, the oxygen plasma operation (15 W, 10 min) was applied once again to remove the unnecessary parts of rGO film, which made the rGO pattern consistent with the microfluidic channel for further research.

### Preparation of rGO-Glass-Based Microfluidic Chip

**Figure 1:**
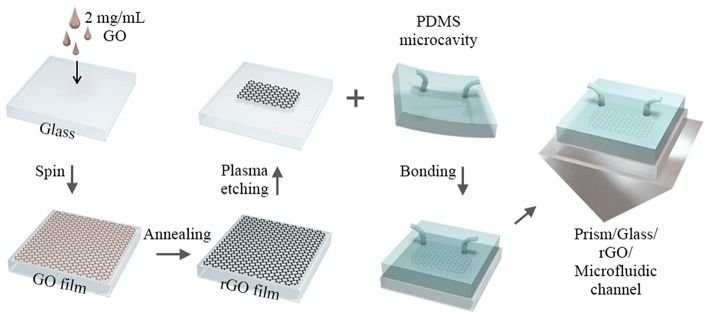
The schematic of the experimental procedure for preparation of the rGO-glass-based microfluidic chip.

The polydimethylsiloxane (PDMS) pre-polymer was firstly prepared with a well-blending between silicone elastomer base and the curing agent (10:1). After its solidification on a template at 75°C for 2 h, a PDMS microfluidic channel with a typical chamber size of 6 × 4 × 0.05 mm^3^ was obtained, with the diameter of the channel about 10 μm. Then, the aforesaid patterned rGO glass and PDMS microfluidic channel were placed in oxygen plasma again to activate the surfaces of PDMS and glass preparing for permanent bonding. Finally, the PDMS microfluidic channel was aligned and bonded with the patterned rGO glass to form the rGO microfluidic chip, which would be further adhered on a rectangular prism with RI matching fluid (*n*_*e*_ = 1.58, IMMOIL-F30CC, OLYMPUS). The top-down materials are PDMS microfluidic channel, rGO, glass, and prism, respectively. The detailed preparation process of the microfluidic device is shown in Figure 1.

## Results and Discussion

### Characterization of rGO Film

**Figure 2:**
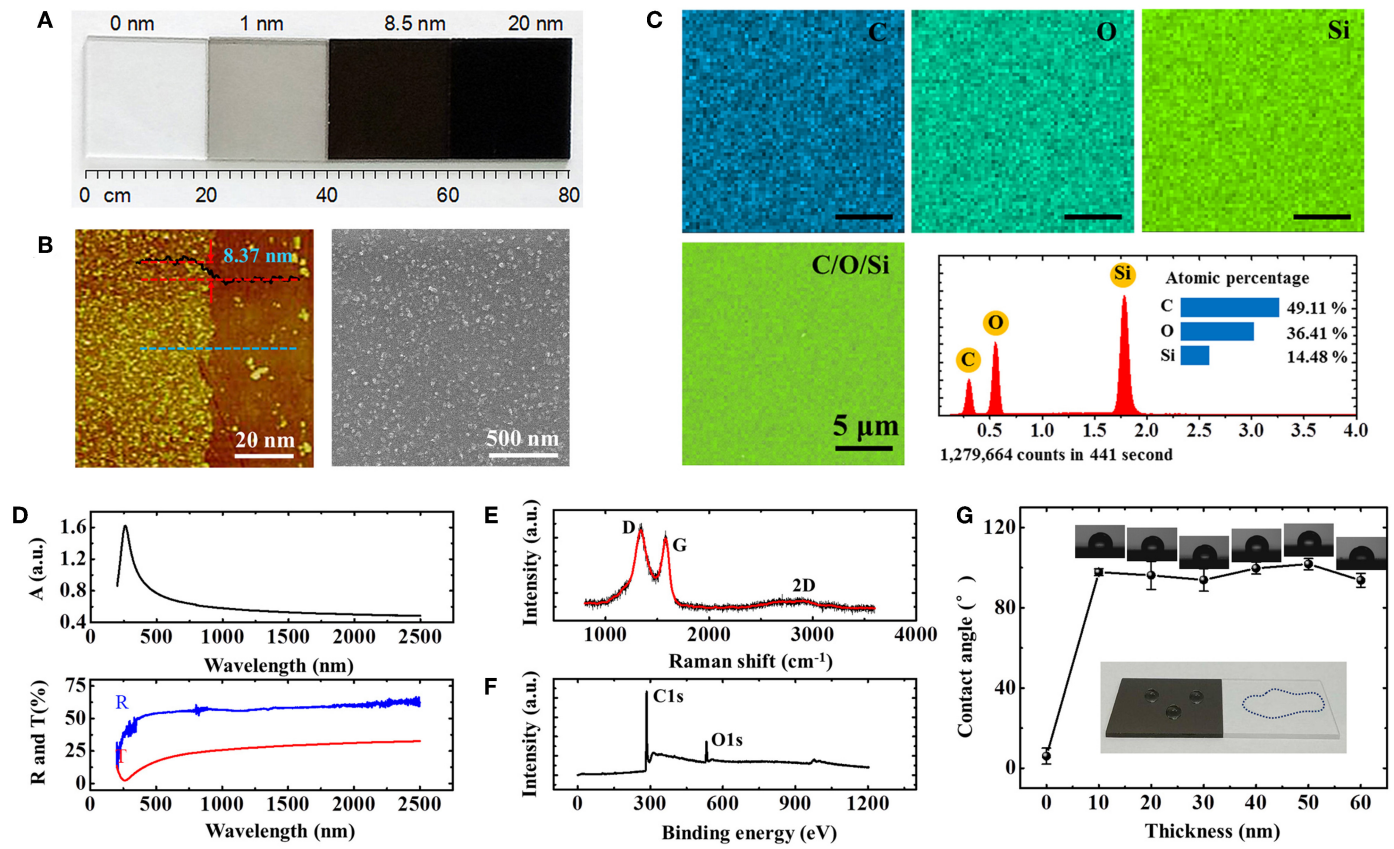
**(A)** Optical images of rGO glass with different thickness of rGO film. **(B–F)** AFM, SEM images, EDS elemental mapping, absorption, transmission, reflection, Raman, and XPS spectra of rGO film with 8.37 nm thickness. **(G)** Different hydrophobic behaviors of bare and rGO glasses. Insert: the optical images of hydro-phobic and -philic properties of the 8.37 nm rGO and bare glass, respectively.

Figure 2A shows the optical images of rGO glass with different thicknesses of rGO film. The transmittance decreases gradually as the thickness increases. The detailed characterizations for a specific thickness (~8.5 nm) have been performed below. Figure 2B exhibits the results of atomic force microscopy (AFM, Veeco, Dimension 3,100 microscope, left panel) and the high-resolution scanning electron microscope (SEM, Phenom ProX) images (right) of the rGO glass. The average thickness of about 8.37 nm with clear altitude difference between the bare glass and rGO film can be observed. The slight fluctuation in height is likely related to the roughness level of common glass substrate. From the SEM image within the field of view of micrometers, it can be seen that the rGO film is actually densely stacked by graphene nanosheets (~100 nm). The strong shear force of gas flow and quick exhaust oxygen in the vacuum system during preparation ensures the smooth and uniformity of rGO film (Lu et al., 2004). Furthermore, the energy-dispersive X-ray spectroscopy (EDS) images are also measured (Figure 2C). The uniform elemental mapping of carbon, oxygen, and silicon can be obtained, respectively. No doping by other chemical elements illustrates that the uniform crystal structure of rGO is not damaged during the preparation (Ismach et al., 2010). In addition, we have also measured the absorption, transmission and reflection spectra of rGO film (Agilent, Cary 5000), as shown in Figure 2D. On one hand, a strong ultraviolet absorption peak at about 260 nm caused by electronic conjugation of sp^2^ carbon can be found, indicating the rGO has a higher reducibility; On the other hand, the film exhibits excellent uniformity of the spectral response within 10% variation cover a very broad spectral range starting from the visible to near-infrared spectrum (~500–2,500 nm) (Nair et al., 2008). The uniformity in both crystal structure and spectral response can completely guarantee the accuracy and the precision of liquid pressure measurements in rGO-glass-based microfluidic chips.

Moreover, for element characteristics analysis, the Raman spectrum (Horiba, LabRAM HR Evolution), and X-ray photoelectron spectroscopy (XPS, Shimadzu, AXIS Supra spectrometer) of rGO film were also measured, as shown in Figures 2E,F. From the Raman spectrum, two peaks at about 1338.6 and 1582.8 cm^−1^ are obviously observed, which, respectively, are signatures of $A_{g}^{1}$ and *E*_2*g*_ phonon modes in graphene corresponding to the D and G bands (Ferrari et al., 2006). The slight enhancement of D/G intensity ratio of rGO film (I_D_/I_G_ = 1.07) means the decreasing size of graphene domains (Han et al., 2015, 2018). However, the degree of reduction of rGO is still relatively large and the conclusion can be drawn by XPS results. Figure 2F shows the XPS characteristics of 8.37 nm rGO film excited under 1486.6 eV and 150 W, highlighting that it is composed of the elements carbon (C1s, 284.85 eV) and oxygen (O1s, 532.55 eV). The C/O atomic ratio is about 11, higher than that of GO, indicates that most of the oxygen functional groups are successfully removed.

At last, the hydrophobic behaviors of rGO glasses are measured because of the microfluidic applications. The hydrophobic properties of the substrate have a significant impact on the accuracy of the microfluidic measurement results. The better the hydrophobicity of the substrate material, the smaller the experimental error caused by the viscous resistance generated between liquid and substrate in microfluidic channels. Here, the static contact angles of bare and rGO glasses are surveyed by hydrophilic angle meter (Xuanyichuangxi, XG-CAMB). As shown in Figure 2G, there is a huge difference in hydrophilicity between bare and rGO glasses. When the surface of common glass is free of rGO, the contact angle was measured about 7.7°, indicating the superhydrophilic property. But when the surface is covered with a layer of rGO, the contact angle was rapidly increased to about 90°. As the rGO thickness increases from 10 to 60 nm, the hydrophobicity does not change too much. The rangeability of contact angle is < 10°. This property is somewhat different from the CVD graphene glass grown by plasma-enhanced CVD system with pure CH_4_ as the precursor (Chen et al., 2017). We infer that the rGO film remains partially hydroxyl and epoxy functional groups at the sheet edges which may slightly weaken the hydrophobicity of rGO glass. However, the stable property of rGO glass also provides a reliable sensing layer for microfluidic chip sensors. This is one of the important reasons why rGO have been always selected as microfluidic sensing materials (Xing et al., 2016; Wang et al., 2018).

### Theoretical Analysis of Reflection Effect of Multilayer Structure

**Figure 3:**
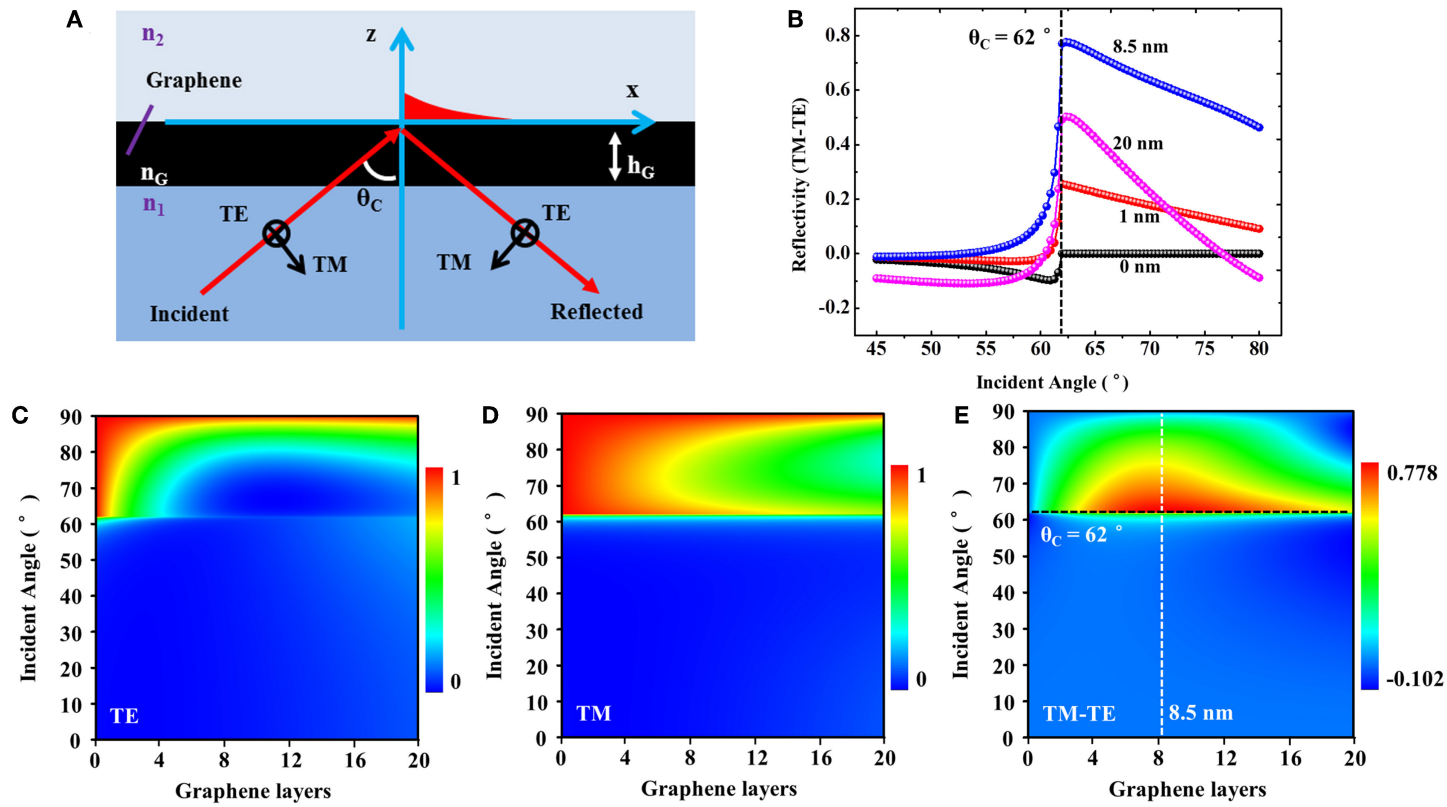
**(A)** The principle of the polarization-sensitive optical absorption effect of rGO. **(B)** The simulation of reflection difference between TM and TE modes (TM-TE) of rGO with different thicknesses. The maximum is about 0.8, which corresponds to the thickness of 8.5 nm and incident angle of 62°. **(C–E)** The detailed reflection map of rGO with different thicknesses and incident angles.

Graphene-based optical sensors were successfully demonstrated to possess high sensitivity in detecting a wide range of RI change for the media, such as gases, liquids, and biomolecules (Xing et al., 2015). Here, we develop a theoretical model of rGO-galss-based microfluidic sensor as a sandwiched structure under different polarized irradiations, which is composed of a high-index medium (common glass, *n*_1_ = 1.51), a low-index medium of fluid (water, *n*_2_ = 1.33), and an rGO layer (*n*_*G*_ = 2.6+1.10 *i*). The rGO layer inserts between *n*_1_ and *n*_2_ and the RI has been confirmed to have a complex optical constant *n*_*G*_ = *n*+*ki*, where *n* is a real part of RI for graphene and *k* is its extinction coefficient. The media of glass and water are considered as two semi-infinite dielectrics and the thickness of rGO (h_G_) is relative to the number of graphene layers (Blake et al., 2007). The detailed schematic of this principle is shown in Figure 3A. Under the sandwiched reflective coupling structure, a fraction of the incident energy can penetrate through the interface between medium *n*_1_ and *n*_2_, and couple to medium *n*_2_. Then, this part of energy propagates along this interface and has strong interaction with rGO layer. When the low-index medium (*n*_2_) changes in RI, the strong interaction between this part of energy and rGO is sensitive to RI changes. This optical signal which is sensitive to the RI can be detected by the reflected light.

The general theory regarding refraction and reflection is governed by the Fresnel's law and Maxwell's boundary conditions, which states that the tangential components of the electric (E) and magnetic (H) fields are continuous across boundaries. Based on the boundary conditions and Snell's law, the incident light was not completely reflected and the energy loss of TE light is greater than that of TM light. The detailed intensity relationship between the incident (i), the reflected (r), and the transmitted light (t) in TE mode is given as follows (Xin et al., 2016):

(1) $$\begin{array}{l} \left(\begin{array}{c} i\\ r \end{array}\right)=\frac{1}{4}\left[\begin{array}{ll} 1+\frac{k_{Gz}}{k_{1z}}\gamma_{1G} & \left(1-\frac{k_{Gz}}{k_{1z}}\gamma_{1G}\right)e^{ik_{Gz}h_{G}}\\ 1-\frac{k_{gz}}{k_{1z}}\gamma_{1G\;} & \left(1+\frac{k_{Gz}}{k_{1z}}\gamma_{1G}\right)e^{ik_{Gz}h_{G}} \end{array}\right]\\ \;\left[\begin{array}{l} \left(1+\frac{k_{2z}}{k_{Gz}}\gamma_{G2}\right)e^{-ik_{Gz}h_{G}}\\ 1-\frac{k_{2z}}{k_{Gz}}\gamma_{G2} \end{array}\right]\left(t\right), \end{array}$$

Where *k* is the wave-vector in the medium and *k*_*z*_ is the corresponding component in z-direction. $k_{Gz}=\sqrt{n_{G}^{2}\cdot k_{0}^{2}-k_{x}^{2}}$, $k_{1z}=\sqrt{n_{1}^{2}\cdot k_{0}^{2}-k_{x}^{2}}$, $k_{2z}=\sqrt{n_{2}^{2}\cdot k_{0}^{2}-k_{x}^{2}}$, $k_{0}=\frac{2\pi }{\lambda_{0}}$. The component in x-direction is *k*_*x*_ = *n*_1_*k*_0_sinθ_1_, where θ_1_ is the incident angle. γ is the relative permeability ratio. $\gamma_{1G}=\frac{\mu_{1}}{\mu_{G}}$, $\gamma_{G2}=\frac{\mu_{G}}{\mu_{2}}$, where μ is the relative permeability. Analogously, we can also use the similar formula to calculate the effect of sandwiched structure on TM mode. The only difference is that we need to change the parameter relative permeability μ to relative permittivity ε.

The simulation of polarization-dependent reflection difference between TM and TE modes (TM-TE) of rGO at an incident wavelength of 633 nm is shown in Figure 3B. As previous report, an optimized value of 8.5 nm can be obtained when the thickness increases (Xing et al., 2014). This is why we mainly focus on this thickness in aforementioned characterizations. Furthermore, we have simulated the reflectivity of rGO with different thicknesses under different incident angles of TE and TM light in detail, as shown in Figures 3C–E. Being near the critical angle θ_c_, there will be a significant enhancement in reflection difference. Therefore, the optimal conditions of *h*_G_ = 8.37 nm and incident angle near the critical angle θ_c_ = 62° are confirmed for the optical detection. Since the RI of rGO varies little over a broad range (~300–1,700 nm), the polarization-dependent properties of rGO can be proven to be a common phenomenon in theory, which suggests that our experimental process can also be extended to the optical systems under other wavelengths (Weber et al., 2010; Zheng et al., 2015) (Supplementary Material S1).

In addition, it should be also noted that only the thickness and RI of the material are considered in the aforesaid model, so theoretically our experiments are not limited by changes in the substrate and liquid environment. However, in the experiment, when the material is in contact with the liquid, problems such as chemical stability and wettability between them present. Material replacement will also have a corresponding impact on the experimental results (Supplementary Material S2).

### Optical Detection of Water Pressure in Microfluidic

**Figure 4:**
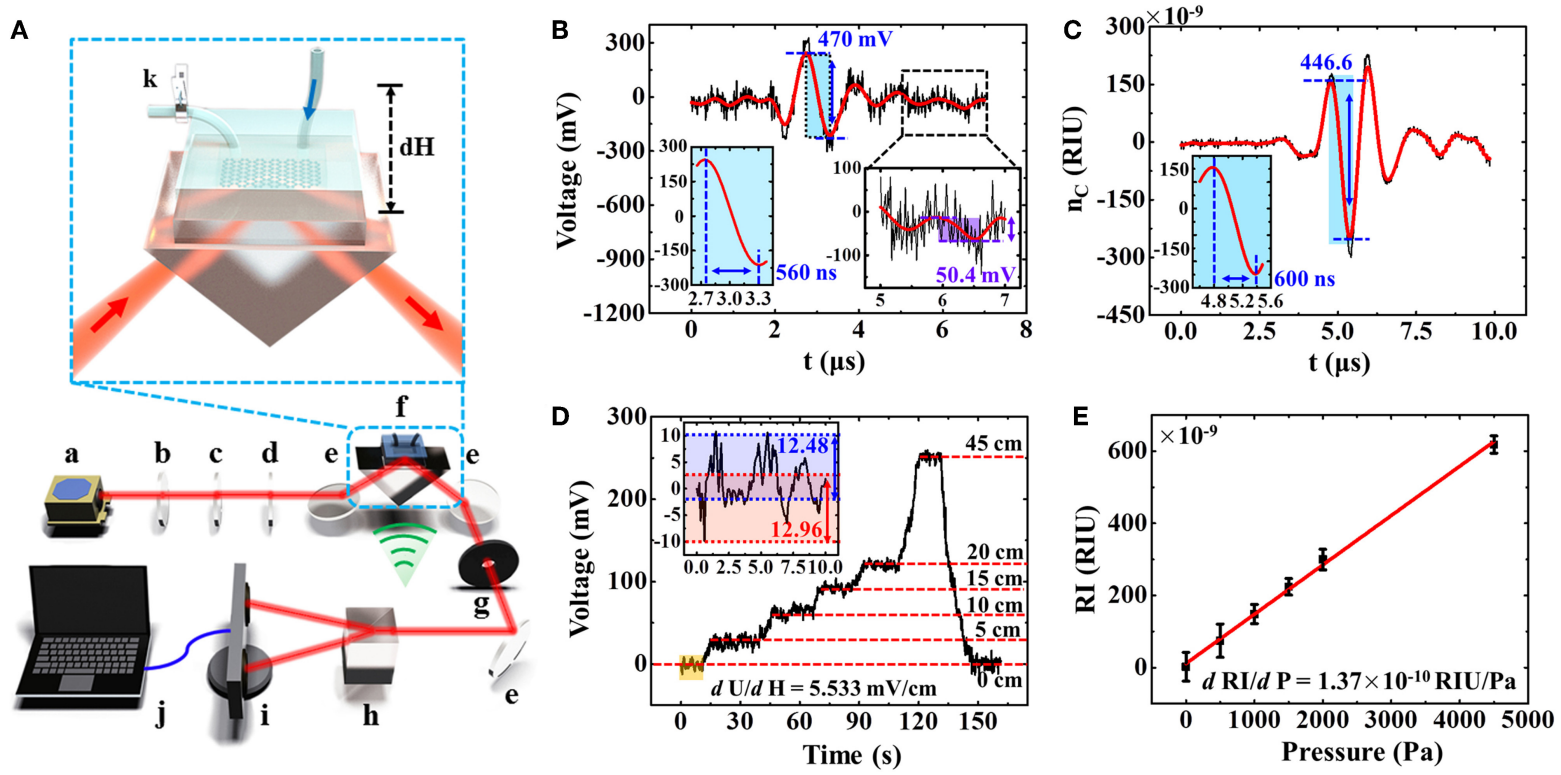
**(A)** Experimental setup schematic of rGO-based microfluidic sensor. a, laser; b, attenuator; c, polarizer; d, 1/4 wave plate; e, reflecting mirror; f, rGO-glass-based microfluidic chip device; g, aperture; h, Wollaston prism; i, balanced detector; j, computer; k, ball valve. **(B)** The direct detection of a weak RI change of water solution generated by an ultrasonic wave. **(C)** The optimized RI change detection of water. **(D)** The relationship between voltage signals and the liquid level. **(E)** The detected RI change as a function of water pressure in the detection window, which is caused by liquid level difference.

A schematic of experimental setup for the rGO-based microfluidic chip with polarization-dependent optical system is shown in Figure 4A. A continuous He-Ne laser (632.8 nm) was chosen as a light source and its beam passed through the attenuator, polarizer, and 1/4 wave plate, respectively. After that, a stable and power-controllable circularly polarized laser can be obtained, which will be further focused onto the center of the microfluidic chip. The lateral beam size the laser spot was 2.25 mm^2^, smaller than that of the microfluidic chamber (6 × 4 × 0.05 mm^3^), which can avoid the disturbance of the information carried by the light source. The way of light-rGO coupling is more important here, so the small changes in parameters of chips, such as the channel diameter and chamber size, have little effect on experimental measurement results (Wang et al., 2018).

Subsequently, the laser reflected from the prism was separated into TE and TM modes with the same optical path by Wollaston prism. The optical path difference should be equal and it has been demonstrated to suppress the common-mode noise and effectively improve the sensitivity of rGO-glass-based microfluidic sensor (Wang et al., 2018). A balanced detector was used to detect the light intensities of TE and TM modes, and then the light information is converted into the electrical signals. With adopting this method, the influence of the laser output instability can be greatly eliminated, and the tiny changes in reflection difference between TM and TE modes caused by the variation of RI in microfluidic environment can be captured and recorded in an accurate way. Therefore, in our experiment, when the pressure of liquid (water, *n*_2_ = 1.33) in microfluidic channel changes, the RI of the medium will be varied accordingly, and it can also be measured by the photo detector (Xing et al., 2014; Wang et al., 2018).

However, in order to further eliminate the influence of other factors, such as the instability of laser intensity and the environmental disturbances, and improve the accuracy and sensitivity of the detection, the weak ultrasonic waves with fixed frequency (1 MHz) were applied to the liquid medium (water, *n*_2_ = 1.33), as shown in Figure 4A. The ultrasound probe has only contact with water and the ultrasound is transmitted to the surface of graphene through the water. Through a modulation of the light-rGO coupling with a periodic, real-time acoustic operation, the alternating pressure of the wave provides informative RI changes of water, thereby improving the extraction of photoelectric signals (Wang et al., 2015). This facilitates an increase in sensitivity to the device's pressure response. The relationship between the RI change (d_n_) and pressure (P) of ultrasonic in water was reported as (Sigrist, 1986):

(2) $$\begin{array}{r} dn/dP=1.35\times 10^{-10} \end{array}$$

When we controlled the pressure of the ultrasonic wave to the water at 1 kPa here, a change in the RI of the response of 1.35 × 10^−7^ could be obtained. Figure 4B shows the variation of the water RI as a function of time under the interaction of single-pulse ultrasonic wave. The ultra-small RI change recorded as a fluctuation of about 470 mV can be observed. The noise (N_noise_) is about 50.4 mV and the signal-to-noise ratio is 9.3. The frequency of ultrasonic wave is set as 1 MHz and the response time is about 560 ns, which indicates quite sensitive response consistent with the frequency of the applied ultrasonic wave here.

Using the improved method described above, we can also confirm the detection limit (D) and sensitivity (S) of the microfluidic sensor. The detection limit can be described as the minimum detectable change in RI, and the relationship between detection limit, sensitivity, and RI change are as follow (Sigrist, 1986):

(3) $$\begin{array}{r} D=N_{noise}/S \end{array}$$

(4) $$\begin{array}{r} S=dU/dn \end{array}$$

where *dU* is the voltage signal variation caused by the RI change. Based on the Equations (3, 4), the detection limit and sensitivity of the rGO-glass-based microfluidic sensor are calculated as 1.4 × 10^−8^ and 3.5 × 10^9^ mV/RIU, respectively. When we enlarge the incident laser intensity, the sensitivity will increases accordingly but the detection limit can be still maintained at the order of 10^−8^ RIU due to the accompanying enhanced noise signal. However, after optimizing the optical path by ultrasonic wave application, the detection process becomes more efficient and accurate, which is more suitable for sensitive and real-time measurement without complex operations such as parameter corrections (D'Amico and Di Natale, 2001). In this letter, to facilitate the experimental operation and improve the signal-noise ratio, a high-pressure ultrasonic wave (~3 kPa), and a weak incident light (~0.1 mW) were used. The high RI change about 4.46 × 10^−7^ RIU (refractive index unit) of the water solution with ultrafast response time (~600 ns) and low signal-to-noise ratio (~23) was obtained, as shown in Figure 4C. Therefore, the response time limit is about 100 ns.

After the optimization of the experimental parameters and the photoelectric signal processing, the RI or voltage changes caused only by the ultrasonic wave can be obtained, which can be used as the baseline for fast measurement of water pressure change. At this point, when the RI of water changes again affected by a tiny and stable pressure in microfluidic channel, a fluctuation of voltage caused by the accompanying RI change will be further detected accurately. For the sake of convenience, the stable water pressure generated here just results from the change of water level height in external hose, and the water pressure in the detection window increased with that. An insert in Figure 4A shows the schematic of this experimental process. Firstly, the typical deionized water was injected into the microfluidic channel and flowed through the micro chamber from right to left. A ball valve was installed at the outlet and placed in closed state. Then placed the water level in external hose at a certain height at the inlet, the rGO-glass-based microfluidic sensor could obviously measure the change of voltage signals. The height difference between the water level away from the detection window is dH. The relationship between pressure (dP) and liquid level height is generally described as Pascal's law:

(5) $$\begin{array}{r} dP/dH=\rho g \end{array}$$

where ρ is a density of the liquid and *g* for a gravitational acceleration. The relationship between the water pressure in microfluidic and the voltage signal is thus established. The detailed relations among the various parameters, such as the liquid level, voltage signal, RI change, external pressure, are listed in Table 1. All of them exhibit in a manner of the linear regularity. Here, the liquid level was placed at 0, 5, 10, 15, 20, 45 cm, which correspond to the voltage signals of 0.91, 30.41, 60.80, 91.76, 122.78, and 253.45 mV, respectively, as shown in Figure 4D. The insert exhibits the fluctuation with about 12.72 mV voltage signal without external pressure. By averaging the test signals at different pressures, we can infer that the detection accuracy limit is about 280 Pa. The relationship between the tiny stable pressure and accompanying RI change can also be derived, as shown in Figure 4E. A linear dependence between the RI change and pressure with the variation factor of 1.37 × 10^−10^ RIU/Pa can be obtained. It should be noted that if the applied pressure changes greatly, due to the change of the internal stress distribution in microfluidics and the exponential relationship between the RI and the reflectance difference (TM-TE), the linear relationship may be destroyed (Xing et al., 2014). Limited by the equipment, the maximum pressure that our devices can measure is about 0.178 Mpa.

**Table 1:** The relationship among height, voltage, RI, water pressure.

**Height (cm)**	**Voltage (mV)**	**RI (RIU)**	**Pressure (Pa)**
0	0.91	2.22 × 10^−9^	0
5	30.41	7.42 × 10^−8^	500
10	60.80	1.48 × 10^−7^	1,000
15	91.76	2.24 × 10^−7^	1,500
20	122.78	2.99 × 10^−7^	2,000
45	253.45	6.18 × 10^−7^	4,500

## Conclusions

In summary, by combining with the reflection coupling structure and ultrasonic waves operation, we developed an ultra-sensitive and real-time method for the detection of water pressure in microfluidics based on a high-performance rGO glass and a smart optical sensing system. The detection limit and sensitivity of this microfluidic sensor are calculated to be 1.4 × 10^−8^ and 3.5 × 10^9^ mV/RIU, respectively, which ensures that the small RI change caused by a pressure as tiny as 500 Pa with a linear variation tendency can be measured directly. The detection accuracy and response time limits are derived to be about 280 Pa and 100 ns, respectively. The ultra-sensitive smart sensor also exhibits a broadband for the frequency change detection of water pressure, which is from static state to more than 1 MHz. For the first time, this rGO sensor demonstrates the importance of liquid pressure on the detection in high-precision microfluidic devices, which may further open up new platforms for designing other measurement variations in more complex environments.

## Data Availability

All datasets generated for this study are included in the manuscript and/or the Supplementary Files.

## Author Contributions

WX, FX, and CG conceived the idea and designed the experiments. WX, TW, and FX fabricated the RGO sensors. TZ, YW, and WJ made the characterizations. TW performed the detections. WX, FX, and CG contributed to data analysis and interpretation. WX, JY, and FX wrote the paper. All authors discussed the results and commented on the manuscript.

### Conflict of Interest Statement

The authors declare that the research was conducted in the absence of any commercial or financial relationships that could be construed as a potential conflict of interest.
