# Supplementary material for: Ultrasensitive Optical Detection of Water Pressure in Microfluidics Using Smart Reduced Graphene Oxide Glass
Source: Front Chem. 2019 May 31;7:395. doi: 10.3389/fchem.2019.00395 (PMC6555094; doi:10.3389/fchem.2019.00395)
Supplement: Supplementary file 1 [file Presentation_1.pdf]

Ultrasensitive optical detection of water pressure in microfluidics using smart reduced graphene oxide glass

Wei Xin[1#], Tiange Wu[2#], Tingting Zou[1], Ye Wang[1], Wenshuai Jiang[3], Fei Xing[2*], JianJun Yang[1], Chunlei Guo[1,4*]

# These authors contributed equally to this work

[1]The Guo China-US Photonics Laboratory, State Key Laboratory of Applied Optics, Changchun Institute of Optics, Fine Mechanics and Physics, Chinese Academy of Sciences, Changchun, China

[2]School of Physics and Optoelectronic Engineering, Shandong University of Technology, Zibo, China

[3]School of Biomedical Engineering, Xinxiang Medical University, Xinxiang, China

[4]The Institute of Optics, University of Rochester, Rochester, New York, USA

*** Correspondence:**

Fei Xing

xingfei@sdut.edu.cn;

Chunlei Guo

guo@optics.rochester.edu

Supplementary Material

S1. Universal applicability of optical detection to laser wavelength

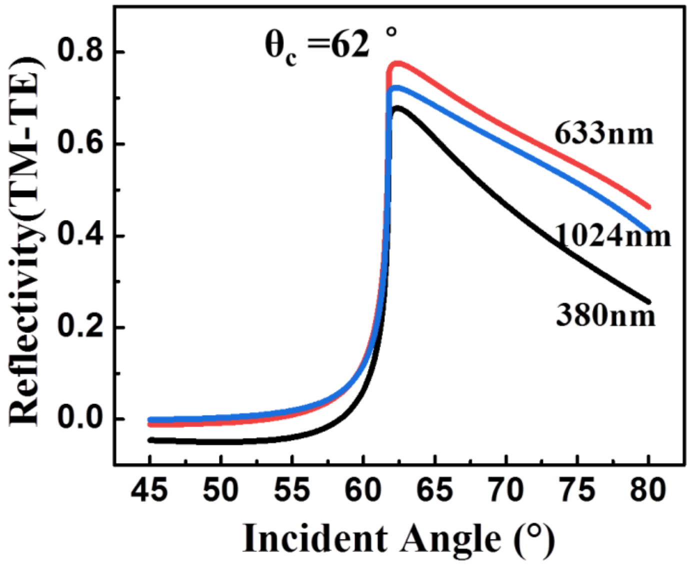

Figure 1. The simulation of reflection difference between TM and TE modes (TM-TE) of rGO under the irradiation of different wavelength lasers

S2. Universal applicability of optical detection to different liquids

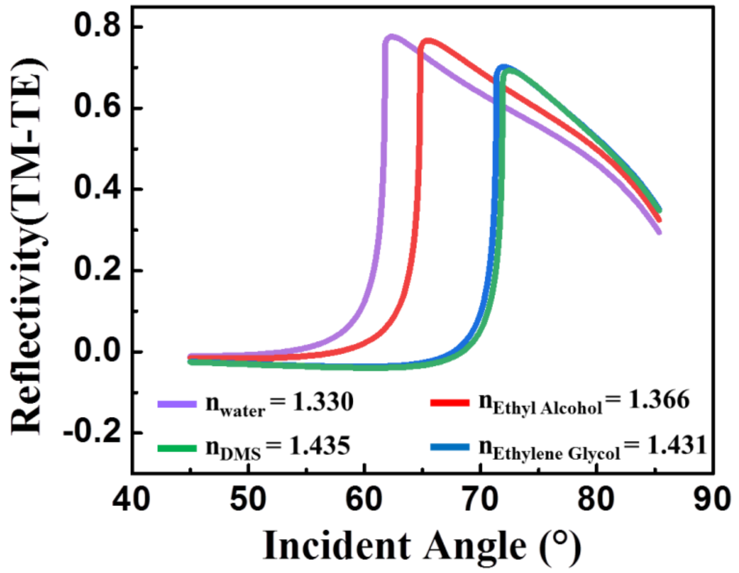

Figure 2. The simulation of reflection difference between TM and TE modes (TM-TE) of rGO with different medium (n_2)
